# Epigenetic Regulation in Hepatocellular Carcinoma Requires Long Noncoding RNAs

**DOI:** 10.1155/2015/473942

**Published:** 2015-03-10

**Authors:** Laura Amicone, Franca Citarella, Carla Cicchini

**Affiliations:** Istituto Pasteur-Fondazione Cenci Bolognetti, Dipartimento di Biotecnologie Cellulari ed Ematologia, Sezione di Genetica Molecolare, Sapienza University of Rome, Viale Regina Elena 324, 00161 Rome, Italy

## Abstract

Recent evidence has proven the relevance of epigenetic changes in the development of hepatocellular carcinoma (HCC), the major adult liver malignancy. Moreover, HCC onset and progression correlate with the deregulation of several long noncoding RNAs (lncRNAs), exhibiting great biological significance. As discussed in this review, many of these transcripts are able to specifically act as tumor suppressors or oncogenes by means of their role as molecular platforms. Indeed, these lncRNAs are able to bind and recruit epigenetic modifiers on specific genomic loci, ultimately resulting in regulation of the gene expression relevant in cancer development. The evidence presented in this review highlights that lncRNAs-mediated epigenetic regulation should be taken into account for potential targeted therapeutic approaches.

## 1. Introduction

Mammalian genomes produce thousands of long nonprotein coding transcripts currently referred to as long noncoding RNAs (lncRNAs) [1, 2]. These RNAs, in many cases expressed from RNA polymerase II promoters, spliced, and polyadenylated, form an extremely complex and heterogeneous class of molecules with a length greater than 200 nt, which distinguishes them from the small noncoding RNAs [3]. These last RNAs include several RNAs, well characterized for their structural and regulatory functions: small nuclear RNAs (snRNAs), small nucleolar RNAs (snoRNAs), microRNAs (miRNAs), piwi-interacting RNAs (piRNAs), small interfering RNAs (siRNAs), and others.

Wide range analysis of cellular transcription by deep sequencing unveiled a large and continuously expanding number of lncRNAs. The GENCODE consortium in the framework of ENCODE (encyclopedia of DNA elements) project estimated, already in 2012, the human catalog of lncRNAs comprising 9277 manually annotated genes and producing 14880 transcripts [4].

LncRNAs can exhibit subcellular localization in precise compartments and, although they are expressed in lower amount with respect to mRNA [4], these transcripts are even more cell-type specific and strictly associated with developmental stages [5–7].

In the last years, increasing evidence showed that lncRNAs do not represent a “transcriptional noise,” having instead great biological significance.

These transcripts, in fact, play a key role in various cellular contexts and are involved in almost every stage of gene expression, in both physiological and pathological cellular conditions. Different lncRNAs control epigenetic processes, such as expression of specific genes, as well as imprinting, and chromosome dosage-compensation, and also transcription, splicing, transport, and translation [8]. Thus, lncRNAs studies have attracted increasing attention, currently representing a top field in the cell biology. Several databases (e.g., lncRNASNP [9], NONCODE [10], LNCipedia [11], lncRNAtor [12], lncRNAdb [13], lncRNAMap [14], and LncRNADisease [15]) collect and make possible the integration of data regarding gene sequences, SNP profiles, expression, and biological activities of many lncRNAs from different sources.

LncRNAs may fold acquiring modular domains with complex tridimensional structures able to bind and guide protein effectors and regulators to specific targets. In particular, a large proportion of known lncRNAs triggers the recruitment of DNA and/or histone modifying complexes on site-specific chromatin contexts, by acting in* cis* (at the site of transcription) or* in trans* (at distantly located genes) ([16], for review [17]).

LncRNAs often display either tumor suppressor or oncogenic activities that frequently have to be ascribed to their capacity to control gene expression by acting at epigenetic level.

In this review, we focus on lncRNAs involved in the epigenetic modifications influencing onset and progression of hepatocellular carcinoma (HCC). Firstly, we summarize the state of the art of research on DNA and histone epigenetic modifications in HCC; secondly, we discuss the biological roles and the molecular functions of known chromatin-associated lncRNAs whose expression is deregulated in HCC stages, highlighting that lncRNAs activities in epigenetic regulation should be taken into account for potential therapeutic approaches.

## 2. HCC and Epigenetics

Levels of chromatin compaction depend on complex mechanisms, including epigenetic modifications that affect either DNA, by methylation and hydroxymethylation of cytosine residues, or histones, by posttranslational additions of several chemical groups (i.e., acetylation, methylation, phosphorylation, ubiquitination, sumoylation, ribosylation, deamination, and proline isomerization). All these posttranslational modifications (PTMs) are tightly controlled by specific enzymes and directly affect chromatin condensation or act as signals for other chromatin-modifying or chromatin-remodeling activities, resulting in transcription regulation [18].

Recent findings point to the involvement of epigenetic mechanisms in the pathogenesis of HCC.

This tumor type represents the major form of adult primary liver cancers and one of the most frequent cancers worldwide. Poor understanding of HCC pathogenesis mechanisms limits diagnosis and treatment at early stages and current therapies, despite recent advances, are essentially unsuccessful. Thus, liver transplantation is still the most efficient treatment, with the lowest risk of tumor recurrence, even if surgical resection and chemoembolization can be valid alternatives in some circumstances [19]. Progression of HCC into a metastatic phenotype implies increased proliferation, cellular dedifferentiation, alterations in the stem/precursor compartment biology, and acquisition of invasiveness by a process of epithelial to mesenchymal transition (EMT) [20, 21].

Apart from several genetic causes, all HCC stages closely correlate to changes in epigenetic patterns of both DNA and histones on several genes crucial for cancer onset and progression.

In line with evidence regarding other tumor types, DNA hypermethylation at specific loci has been correlated to inactivation of tumor suppressor genes also in HCC. For example, epigenetic silencing of Ras pathway inhibitors was found, resulting in Ras and its downstream effectors ERK, AKT, and RAL activation. Similarly, an inactivation of angiogenesis inhibitors has been shown in HCC with poor prognosis [22]. Moreover, changes in gene expression profiles of HCC cells upon treatment with the demethylating agent 5-Aza-2′-deoxycytidine lead Wong and colleagues [23] to identify the Kunitz-type serine protease inhibitor tissue factor pathway inhibitor-2 (TFPI-2) as a new tumor suppressor significantly downregulated in HCCs; TFPI-2 overexpression, indeed, significantly suppressed both proliferation and invasiveness of tumor cells.

Moreover, a wide range analysis of genome methylation in HCC compared with normal liver provided a methylation “signature” useful in diagnosis and prognosis of tumor.

For example, some tumor suppressor genes (i.e., HIC1, GSTP1, SOCS1, RASSF1, CDKN2A, APC, RUNX3, and PRDM2) [24] showed significantly higher methylation levels already in the early HCC, thus suggesting that they could be silenced in the first steps of hepatocarcinogenesis and could represent possible predictive biomarkers. Furthermore, an approach based on analysis of DNA methylation in HCC, gene reexpression in cells after epigenetic unmasking, and subsequent validation identified sphingomyelin phosphodiesterase 3 (SMPD3) and neurofilament heavy polypeptide (NEFH) as tumor suppressor genes differentially methylated in HCC [25]. Hernandez-Vargas and colleagues [26], instead, analyzed a panel of cancer-related gene promoters identifying a set of hypermethylated genes that discriminated between HCC cells and nontumor tissues or other tumor types. Moreover, Song et al. found differential methylation not only of genome CpG islands but also of less-characterized surrounding regions that could have effects on important signaling networks (i.e., cellular development, gene expression, and cell death) [27].

DNA methylation is orchestrated by the DNA methyltransferase (DNMT) enzymes; DNMT1 is mainly involved in the maintenance of established methylation patterns [28], while DNMT3 A and B are the enzymes able to efficiently operate* de novo* methylation of DNA sequences [29]; thus they are required during development and frequently deregulated in cancer cells. DNMTs increase in hepatocarcinogenesis and their upregulation correlates with a progressive increase in the number of methylated genes in HCC with respect to normal liver [30]. Notably, an inverse correlation between the levels of DNMT3A and of microRNA-29 family members, targeting this enzyme transcript, has been found in HCC, with increased DNA methylation associated with aggressiveness of tumor cells [31]. Moreover, DNMT3A overexpression was observed also in HCC cell lines, where its depletion determined demethylation of the tumor suppressor PTEN promoter and correlated with cell proliferation inhibition [32]. Interestingly, HCC cells treatment with a DNMTs inhibitor impaired metastasis [33].

Several studies also revealed the correlation between HCC and aberrant histone modifications.

Chromatin accessible to transcriptional factors is generally characterized by the acetylation of histone tails controlled by histone acetyltransferases (HATs). Concerning, instead, histone methylation, its effects depend on (i) which amino acid residue is methylated and (ii) which is the methylation form, because one, two, or three methyl groups (indicated as me1, me2, and me3, resp.) can be dynamically added to the histone tail. In particular, the trimethylation on lysine 27 of histone H3 (H3K27me3) is a key repressive mark.

Polycomb group proteins (PcG) are the main regulators of repressive epigenetic modifications of chromatin, initiating and maintaining the transcriptional repression of target genes. These proteins are classified in two groups, the polycomb repressive complexes (PRC) 1 and 2, taking into account the multimeric complexes with which they associate. PRC2, composed of Eed, Suz12, Ezh2, and RBBP7/4, initiates gene silencing by methylation of histone H3 lysine 27 (H3K27); PRC1, composed of Ring1a/1b, Nph1, and Bmi1, maintains gene silencing by monoubiquitinating histone H2A lysine 119 (H2AK119) [34].

In a recent study, a number of HCC was characterized for both acetylation and trimethylation state of H3K27 [35] and a correlation between high levels of H3K27ac and H3K27me3 and an aggressive behaviour has been identified. Other studies associated also high level of H3K4me3 with poor HCC prognosis [36, 37]. In addition, the hyperacetylation of histone H3 on lysine 9 (H3K9ac) and of histone H4 on lysine 8 (H4K8ac) in HCC compared to cirrhotic and normal livers was reported [38].

In accordance with these findings, the deregulation of some histone modifiers has been observed in HCC, suggesting a key role of these molecules in the pathogenesis of the tumor. In particular, members of PRC2 and PRC1 complexes are frequently deregulated in tumor cells and the consequent aberrant gene expression drives progression of hepatocarcinogenesis towards metastatic stages. Among PRC subunits, considerable evidence concerns the methyltransferase Ezh2, directly responsible for H3K27 trimethylation. Invasive properties of HCC tumors were strongly associated with Ezh2 upregulation [39, 40]; consistently, Ezh2 levels were proposed as diagnostic biomarkers for the detection of HCC in liver biopsies [41]. Ezh2 and its associated partners in PRC2, indeed, were shown to be responsible for the silencing of several microRNAs known for their role as tumor suppressors (i.e., miR-139-5p, miR-125b, miR-101, let-7c, and miR-200b), thus enhancing liver cells motility and metastasis [42]. Notably, one of these microRNAs, miR-101, was recently characterized as a negative regulator of the PRC2 complex subunits Ezh2 and Eed; thus a double-negative feedback loop exists and is deregulated in HCC [43].

Moreover, Ezh2 was found to activate the *β*-catenin signaling by epigenetic repression of different negative regulators of Wnt pathway (e.g., AXIN2, NKD1, and PRICKLE1) implying a control on cellular proliferation; downregulation of Ezh2, indeed, reduced HCC cell growth [44].

Concerning the subunit of PRC2 complex Suz12, while it appeared upregulated in HCC as well as other PRC2 components [42], its protein levels were instead negatively controlled via phosphorylation in hepatitis B virus (HBV) X protein-mediated transformation of the hepatocyte [45]. Moreover, hepatocyte loss of Suz12 determined the derepression of genes strongly expressed in hepatic cancer stem cells (i.e. EpCAM, BAMBI, DKK2, and DLK1) [46].

With regard to the PRC1 member Bmi1, its deregulation in HCC has been correlated to both early-stage HCC [47] and progression of carcinoma [40, 48].

HCC is also characterized by deregulation of other chromatin modifying enzymes. For example, Patt1, a GNA family acetyltransferase exhibiting proapoptotic activity in hepatoma cells, was downregulated [49]. Tumor cells also exhibited rare dimethylation of lysine 4 of histone H3 (H3K4diMe), probably caused by deregulation of the Ash2 complex, which methylates H3K4, and LSD1 that, conversely, demethylates the same residue [50].

Moreover, in hepatocarcinogenesis of chronic viral hepatitis protein phosphatase 2A (PP2A) and the protein arginine methyltransferase 1 (PRMT1) are both dysregulated [51].

## 3. LncRNAs Involved in Epigenetics of HCC

Deregulation of lncRNAs is related to several human diseases and a broad range of cancers [52]. In different tumors and, in particular, in HCC, several lncRNAs have been characterized as tumor suppressors; otherwise, these regulators may exert oncogenic functions and are overexpressed in correlation with tumor progression, metastasis, and poor patient outcome.

### 3.1. HOTAIR

The HOX TranscriptAntisense Intergenic RNA (HOTAIR) is a polyadenylated and spliced transcript, antisense to the mammalian homeobox transcription factor C (HOXC) locus, identified by Rinn and colleagues [53]. Based on its genomic locus, the lncRNA HOTAIR is further classified as large intergenic noncoding RNA (lincRNA): it lies, indeed, as a distinct transcription unit within the intergenic region encompassed between two different genes [53].

Sequence analyses proved that HOTAIR exists in mammals and evolved faster than nearby HoxC genes [54]. HOTAIR exhibits conserved conformational structures, despite differences in sequences among the species. Murine HOTAIR, indeed, has a first exon significantly conserved with respect to human sequence but the second exon shows high conservation only in a subdomain. Moreover, transcriptome analysis by deep sequencing demonstrated that the murine HOTAIR is likely encoded by two exons, instead of six as in humans [55].

Chiyomaru and colleagues [56] recently reported the HOTAIR interaction with miR-34. In this situation, HOTAIR could act as a sponge, interfering with the miR-34 activities, thus acting as an oncogene. Mir-34, in fact, is considered a tumor suppressor microRNA, regulated by p53 and influencing apoptosis and cell cycle arrest as well as senescence [57]. HOTAIR also sponges miR-331-3p, acting as competing endogenous RNA to regulate the expression of HER2 [58].

However, the fundamental and better-characterized functions of HOTAIR are its roles as “guide” and “scaffold.”

Pertaining to the role of guide, HOTAIR was found to interact through its 5′-end with PRC2 and specifically lead to* in trans* recruitment of this complex on several distantly located genes [59]. The interaction between HOTAIR and the PRC2 subunit Ezh2 is enhanced by the posttranslational phosphorylation of this methyltransferase by cyclin-dependent kinase 1 (CDK1) [60].

The HOTAIR-mediated trans-recruitment is consistent with recent finding that lincRNAs can couple DNA and chromatin modifying activities by binding the enzymes and triggering their specific targeting [16, 53, 61]. HOTAIR, indeed, not only suppresses* in trans* the expression of the HOXD locus on a different chromosome, but also acts in a genome-wide manner, controlling H3K27 trimethylation, then epigenetic silencing, of several target sequences [53].

Regarding the HOTAIR role as scaffold, this depends on its capacity to recruit several chromatin modifiers organizing a molecular platform. Apart from the PRC2 binding at its 5′-end, indeed, HOTAIR binds through its 3′-end the lysine-specific demethylase 1 (LSD1), the enzyme that, complexed with the corepressors REST (RE1-silencing transcription factor) and CoREST (corepressor for element 1-silencing transcription factor), erases the activating H3K4 trimethylation. Thus, HOTAIR assembles and addresses different histone modifiers to chromatin: the positioning of PRC2 and LSD1 complexes on specific chromosomal loci couples the H3K27 trimethylation and the H3K4 demethylation, fundamental chromatin modifications for gene silencing [59] (Table 1). The mechanism of HOTAIR recruitment on specific chromatin regulative regions is still unclear, considering that it is not an antisense for all its targets. As hypothesized for other known lncRNAs, specific transcriptional factors, able to act as both RNA and DNA binding proteins, could be involved in forming a molecular platform in chromatin contexts.

HOTAIR overexpression was proven to promote metastasis and was correlated to poor prognosis of several tumor types, for example, breast [61], colorectal [62], and nasopharyngeal [63] cancers and, particularly, it is overexpressed in HCC tissues and liver cancer cells [64, 65].

Notably, its knockdown significantly affects migratory and invasive properties [64] as well as susceptibility to apoptosis [65] of hepatic cells.

Two recent reports proposed that the role of HOTAIR in tumor progression and acquisition of invasiveness can involve an EMT process. Xu and colleagues demonstrated that HOTAIR knockdown concurrently suppressed invasion and reversed EMT of gastric cancer cells [66]. Alves and colleagues also proved that TGF*β* treatment, inducing EMT, increased HOTAIR levels. Interestingly, HOTAIR silencing by a siRNA approach prevented the transitional program and the acquisition of stem properties by colon and breast cancer cells [67].

Moreover, enforced expression of HOTAIR in epithelial cancer cells determined the retargeting of PRC2 complex causing an occupancy pattern on genome resembling that of fibroblasts, in parallel with metastatic properties acquisition [61]. In spite of this evidence, the mechanism by which HOTAIR can direct an EMT program is still poorly elucidated.

In conclusion, HOTAIR is one of the well-studied PRC2 interacting lncRNAs and its expression appears to be a driving force for the acquisition of malignant properties by HCC, as well as other tumor types. It is able to recruit different players in controlling gene expression, organizing a molecular platform able to specifically retrieve target sequences.

Nevertheless, a comprehensive characterization of HOTAIR mechanism of function is still needed and further studies of its related pathways and partners could be useful to identify new possible therapeutic targets.

### 3.2. H19

The human H19 gene encodes a ~2.3-kilobase long, spliced, and polyadenylated lncRNA [68]. The first exon of H19 gene encodes also for the microRNA675, shown to control placental growth at the end of gestation by targeting the transcript of Igf1r gene [69–71].

The H19 gene belongs to a conserved gene cluster that plays an important role in embryo development and growth control. H19 locus is imprinted in both humans and mice and is expressed from the maternal allele [72]. The cluster also contains the imprinted gene for insulin-like growth factor 2 (Igf2), which is paternally expressed. During embryo development H19 and Igf2 are expressed in endoderm- and mesoderm-derived tissues and their expression is regulated by an intergenic differentially methylated region (DMR), also called imprinting control region (ICR), and by a common enhancer region that promotes either maternal H19 expression or paternal Igf2 expression [73]. H19 lncRNA levels are high during embryogenesis and are downregulated, after birth, in most of adult tissues [74]. The function of H19 has been explored in mice and cell lines by using knockdown and transgenic approaches. It was shown that targeted deletion of maternal H19 locus (H19Δ3) in mice induces an overgrowth phenotype and the upregulation of Igf2 and of several genes belonging to a network of imprinted genes (IGN) involved in the control of embryonic growth. Subsequent transgenic H19 expression downregulates* in trans* the expression of Igf2 and IGN genes and restores the normal phenotype [73, 75]. The mechanisms underlying this regulation have been, at least in part, elucidated by Monnier and colleagues [71]. They demonstrated that H19 lncRNA directly represses the expression of three genes (Igf2, Slc38a4, and Peg1) of the IGN by recruiting the MBD1 protein to the DMRs of these imprinted genes. H19 deletion was associated with a loss of H3K9me3 on Igf2, Slc38a4, and Peg1 DMRs, thus indicating that H19 contributes to the maintenance of a transcriptional repressive mark on its target genes. Therefore, maternally expressed H19 mediates an epigenetic control of paternally expressed IGN genes to control growth of the embryo.

H19 role in tumorigenesis is controversial, since it has been described as either protumorigenic or tumor suppressor depending on the context.

Upregulation of H19 and Igf2 expression has been shown in HBV-associated HCC [76] and an imbalance in H19 and Igf2 expression associated with hepatocellular carcinoma progression [77]. Finally, Sohda et al. [78] reported high levels of H19 in 37% of HCCs compared with nontumorous livers. However, most of these observations do not highlight the functional significance of H19 overexpression in HCCs. Higher H19 levels can simply be the result of altered chromatin structure in liver cancer.

Invasiveness and metastatic potential of tumor cells are associated with the process of EMT and H19 has been demonstrated to influence different players involved in this trans-differentiation phenomenon. Indeed, a significant negative correlation was observed both* in vivo* and* in vitro* between H19 and the levels of the key epithelial adhesion molecule E-cadherin, in which repression is causal to the EMT [85]. H19 was proven to directly contribute to the transcriptional repression of E-cadherin by assisting the binding of the epigenetic regulator Ezh2 to its promoter [85]. Meanwhile, H19 was demonstrated to induce derepression of the Wnt/*β*catenin pathway that positively regulates EMT. In fact, this lncRNA facilitates the recruitment of Ezh2 on the promoter of Nkd1, thus impairing the expression of this inhibitor of the Wnt/*β*catenin signaling. As a result, in bladder cell lines, H19 promotes invasiveness by indirectly activating Wnt/*β*catenin pathway and directly inhibiting E-cadherin transcription [85]. Consistently, in recent studies it has been shown that H19 levels are upregulated in bladder cancer tissues and are higher in patients with invasive bladder cancer compared to patients with noninvasive cancer. Matouk et al. [86] also found high levels of H19 in metastatic sites of biopsies from different primary tumors and demonstrated that, in different cancer cell lines, among which is Hep3B, factors that may induce the EMT process such as TGF*β*, hypoxia and HGF/SF also increase the expression levels of H19 gene. They also observed that ectopic expression of H19 enhanced Hep3B invasiveness* in vitro* and H358 (lung cancer cell line) metastasizing capability* in vivo*, concluding that H19 is involved in enhancing invasion and metastasis [87].

Interestingly, H19 was demonstrated to also play a role in MDR1-associated drug resistance in human hepatocarcinoma cell lines HepG2 and Hep3B. H19 was found overexpressed in doxorubicin-resistant cells and its overexpression correlated with MDR1 promoter demethylation, higher P-glycoprotein levels, and doxorubicin resistance. H19 knockdown leads to MDR1 promoter hypermethylation, decreased expression of P-glycoprotein, and doxorubicin sensitization. As a consequence, H19 may contribute to multidrug resistance in liver cancer cell lines by inducing demethylation of MDR1 promoter and overexpression of P-glycoprotein [88].

Conversely, H19 has been found to regulate the rate of tumor metastasis in advanced stages of HCC via the epigenetic activation of the miR-200 family [79]. The authors, indeed, have shown that H19 expression is significantly lower in invasive HCC cancers compared to noninvasive tumors and that H19 expression positively correlates with miR-200b expression. Knockdown of H19 in HCC cell lines promoted cell invasion capability* in vitro* end* in vivo* while H19 ectopic expression inhibited invasion. Moreover, H19 knockdown decreased the expression of epithelial markers including E-cadherin and increased the expression levels of mesenchymal markers such as N-cadherin, Snail1, Vimentin, and Twist1, thus indicating that H19 could suppress the EMT process that is involved in tumor progression and invasiveness. Indeed, ectopic H19 upregulated the expression of miR-200 family members that play a crucial role in EMT inhibition and whose expression is downregulated in HCC. H19 activated miR-200 family expression by associating with the HnRNPU/PCAF/RNA PolII complex and favoring the binding of the complex and consequent histone H3 acetylation on the promoter of miR-200 family [79] (Table 1).

Remarkably, a tumor suppressor function of H19 has been demonstrated* in vivo* by using three different mice models of tumorigenesis. In SV40 induced hepatocarcinoma model, H19 deletion was associated with shortening of latency and faster appearance of the tumors. Accordingly, in the absence of H19, induced teratocarcinomas were larger and the number of polyps found in APC mutant mice was more than twice higher than in the same tumor models expressing H19 [89]. Moreover, inhibition of lncRNAH19 and miR-675 promoted migration and invasion of human HCC cells by activating the AKT/GSK-3*β*/Cdc25A signaling pathway [90].

We can conclude that, depending on the biological context, during development and disease, H19 was found not only to have multiple functions but also to act on different target genes through distinct molecular mechanisms.

### 3.3. HEIH

lncRNA HEIH has been identified as one of 174 lncRNAs that were differentially expressed between HCC and nontumoral samples. It is a polyadenilated RNA polymerase II-encoded transcript; it is located in both nucleus and cytoplasm of HCC cells and has no homolog in mouse. LncRNA HEIH downregulation correlated,* in vitro*, with reduced proliferation and increased expression of proteins controlling cell cycle progression (i.e., p16, p27, and p21).* In vivo* tumors from lncRNA HEIH-downregulated xenografts showed, indeed, a reduced growth compared with that of tumors formed from controls. lncRNA HEIH has been shown to associate with EZH2 and to increase the binding of EZH2 to p16 and p21 promoters; however, H3K27 levels were increased only across p16 promoter (Table 1). In conclusion, this lncRNA contributes to transcriptional repression of cell cycle controlling genes by different mechanisms [80].

### 3.4. MALAT1

The lncRNA MALAT1, localized in the “nuclear speckles,” regulates (i) the alternative splicing, by interacting with splicing factors so influencing their distribution and levels [81], and (ii) the activation of genes controlling the cellular growth, by interacting with the nonmethylated form of Polycomb 2 protein (Pc2), and hence the sumoylation of E2F1 and the subsequent gene expression [82].

Moreover, the interaction between MALAT1 and methylated or unmethylated Pc2 controls the relocation of cell cycle related genes in PcG bodies or interchromatin granules (ICG), resulting in gene repression or activation [82] (Table 1).

Recent findings correlated MALAT1 to HCC. In particular, Lai and colleagues [91] evaluated the expression of MALAT1 in cancer cell lines and in more than one hundred HCC samples. Together with the upregulation of MALAT1 in both cell lines and clinical tissue samples, the authors observed that patients with high expression level of MALAT1 had a significantly increased risk of tumor recurrence after liver transplantation. Moreover, they reported the effects of inhibition of MALAT1 by RNA interferences in hepatoma cell lines on the reduction of cell viability, motility, and invasiveness and on the increase of the sensitivity to apoptosis indicating MALAT1 as a novel biomarker for predicting tumor recurrence after liver transplantation and as a promising therapeutic target.

### 3.5. MEG3

The maternally expressed gene 3 (MEG3) lncRNA was found strongly downregulated in several (HCC) cell lines and in HCC samples. Its enforced expression in HCC cells counteracts the transformed phenotype by reducing the uncontrolled growth and inducing apoptosis [92].

With respect to its specific role in epigenetic control of gene expression, a recent study unveiled as MEG3, together with other lncRNAs, stimulates the interaction between JARID2, an accessory component of PRC2, and EZH2* in vitro* as well as the JARID2-mediated recruitment of PRC2 to chromatin* in vivo* [83] (Table 1).

### 3.6. HOTTIP

The HOXA transcript at the distal tip (HOTTIP) is a lncRNA significantly upregulated in HCC. Its gene is located in physical proximity to the gene locus HOXA, whose deregulation was, in turn, described in hepatocarcinogenesis. In particular, levels of HOTTIP and HOXA13 were recently related to the clinical progression of HCC and predictive for prognosis [93]. HOTTIP belongs to the family of the “cis-acting” noncoding RNAs (that are embedded in the same genomic loci of their target genes) [94]. In particular, a chromosomal loop brings HOTTIP RNA from its gene into close proximity to its targets. HOTTIP binds the adaptor protein WDR5 that, in turn, recruits the methyltransferase MLL, driving histone H3 lysine 4 trimethylation and gene transcription [84] (Table 1). Moreover, it is conceivable that chromosome looping can extend HOTTIP range of action over large distances.

## 4. Conclusions

The knowledge about mechanisms governing gene expression in tumor onset and progression appears essential for efficient therapies setting.

In HCC, some deregulated lncRNAs are predictive markers for precise tumor stages and, most importantly, they have been characterized as tumor suppressor and/or oncogenes. These functional roles depend on their ability to specifically control gene expression as part of a broad epigenetic regulatory network (Table 1).

More efforts are needed to better elucidate the roles of these molecules in all HCC stages, as well as in other tumor types, but increasing evidence already proved the potential role of these transcripts as targets for possible anticancer treatments, similarly to currently well-utilized microRNAs. Proposed HCC epigenetic drugs are represented by inhibitors of DNMTs [95–97] and histone deacetylases [98, 99] that, by interfering with epigenetic regulators activities, simultaneously affect multiple target genes. Nevertheless, the possible control of the levels of particular lncRNAs, for example, by simple siRNA approaches, could offer the opportunity to modulate epigenetic modifications and, in turn, gene expression, at specific chromosomal loci, thus guaranteeing a more targeted therapeutical intervention.

## Figures and Tables

**Table 1:** List of lncRNAs deregulated in HCC and their role in epigenetic mechanisms of gene regulation.

LncRNA	Epigenetic mechanisms in HCC	References
*HOTAIR *	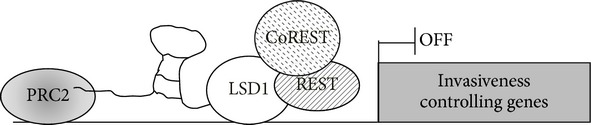	[60–62]

*H19 *	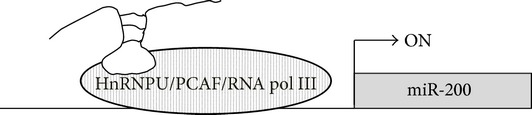	[79]

*HEIH *	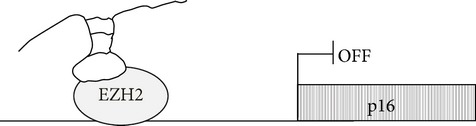	[80]

*MALAT1 *	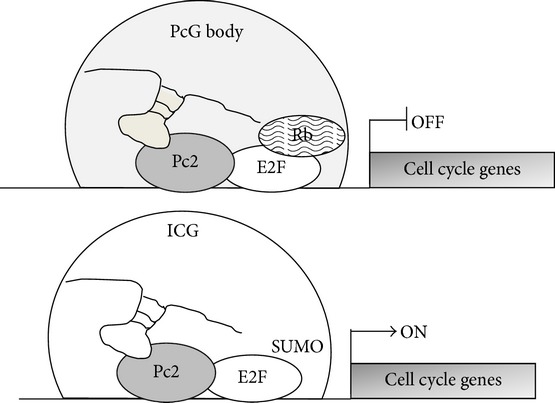	[81, 82]

*MEG3 *	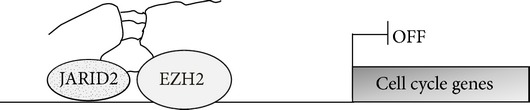	[83]

*HOTTIP *	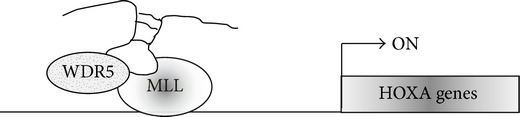	[84]

## References

[1] Carninci P., Kasukawa T., Katayama S. (2005). The transcriptional landscape of the mammalian genome. *Science*.

[2] Wang K. C., Chang H. Y. (2011). Molecular mechanisms of long noncoding RNAs. *Molecular Cell*.

[3] Guttman M., Amit I., Garber M. (2009). Chromatin signature reveals over a thousand highly conserved large non-coding RNAs in mammals. *Nature*.

[4] Derrien T., Johnson R., Bussotti G. (2012). The GENCODE v7 catalog of human long noncoding RNAs: analysis of their gene structure, evolution, and expression. *Genome Research*.

[5] Cabili M., Trapnell C., Goff L. (2011). Integrative annotation of human large intergenic noncoding RNAs reveals global properties and specific subclasses. *Genes & Development*.

[6] Djebali S., Davis C. A., Merkel A. (2012). Landscape of transcription in human cells. *Nature*.

[7] Ravasi T., Suzuki H., Pang K. C. (2006). Experimental validation of the regulated expression of large numbers of non-coding RNAs from the mouse genome. *Genome Research*.

[8] Clark M. B., Mattick J. S. (2011). Long noncoding RNAs in cell biology. *Seminars in Cell and Developmental Biology*.

[9] Gong J., Liu W., Zhang J., Miao X., Guo A.-Y. (2015). lncRNASNP: a database of SNPs in lncRNAs and their potential functions in human and mouse. *Nucleic Acids Research*.

[10] Bu D., Yu K., Sun S. (2012). NONCODE v3.0: integrative annotation of long noncoding RNAs. *Nucleic Acids Research*.

[11] Volders P.-J., Helsens K., Wang X. (2013). LNCipedia: a database for annotated human IncRNA transcript sequences and structures. *Nucleic Acids Research*.

[12] Park C., Yu N., Choi I., Kim W., Lee S. (2014). lncRNAtor: a comprehensive resource for functional investigation of long non-coding RNAs. *Bioinformatics*.

[13] Amaral P. P., Clark M. B., Gascoigne D. K., Dinger M. E., Mattick J. S. (2011). LncRNAdb: a reference database for long noncoding RNAs. *Nucleic Acids Research*.

[14] Chan W. L., Huang H. D., Chang J. G. (2014). lncRNAMap: a map of putative regulatory functions in the long non-coding transcriptome. *Computational Biology and Chemistry*.

[15] Chen G., Wang Z., Wang D. (2013). LncRNADisease: a database for long-non-coding RNA-associated diseases. *Nucleic Acids Research*.

[16] Khalil A. M., Guttman M., Huarte M. (2009). Many human large intergenic noncoding RNAs associate with chromatin-modifying complexes and affect gene expression. *Proceedings of the National Academy of Sciences of the United States of America*.

[17] Mercer T. R., Mattick J. S. (2013). Structure and function of long noncoding RNAs in epigenetic regulation. *Nature Structural and Molecular Biology*.

[18] Chen T., Dent S. Y. R. (2014). Chromatin modifiers and remodellers: regulators of cellular differentiation. *Nature Reviews Genetics*.

[19] El-Serag H. B. (2011). Hepatocellular carcinoma. *The New England Journal of Medicine*.

[20] Thorgeirsson S. S., Grisham J. W. (2002). Molecular pathogenesis of human hepatocellular carcinoma. *Nature Genetics*.

[21] Conigliaro A., Amicone L., Costa V. (2013). Evidence for a common progenitor of epithelial and mesenchymal components of the liver. *Cell Death and Differentiation*.

[22] Calvisi D. F., Ladu S., Gorden A. (2007). Mechanistic and prognostic significance of aberrant methylation in the molecular pathogenesis of human hepatocellular carcinoma. *The Journal of Clinical Investigation*.

[23] Wong C.-M., Ng Y.-L., Lee J. M.-F. (2007). Tissue factor pathway inhibitor-2 as a frequently silenced tumor suppressor gene in hepatocellular carcinoma. *Hepatology*.

[24] Nishida N., Kudo M., Nagasaka T., Ikai I., Goel A. (2012). Characteristic patterns of altered DNA methylation predict emergence of human hepatocellular carcinoma. *Hepatology*.

[25] Revill K., Wang T., Lachenmayer A. (2013). Genome-wide methylation analysis and epigenetic unmasking identify tumor suppressor genes in hepatocellular carcinoma. *Gastroenterology*.

[26] Hernandez-Vargas H., Lambert M.-P., Le Calvez-Kelm F. (2010). Hepatocellular carcinoma displays distinct DNA methylation signatures with potential as clinical predictors. *PLoS ONE*.

[27] Song M.-A., Tiirikainen M., Kwee S., Okimoto G., Yu H., Wong L. L. (2013). Elucidating the landscape of aberrant DNA methylation in hepatocellular carcinoma. *PLoS ONE*.

[28] Pradhan S., Esteve P.-O. (2003). Mammalian DNA (cytosine-5) methyltransferases and their expression. *Clinical Immunology*.

[29] Okano M., Bell D. W., Haber D. A., Li E. (1999). DNA methyltransferases Dnmt3a and Dnmt3b are essential for de novo methylation and mammalian development. *Cell*.

[30] Saito Y., Kanai Y., Sakamoto M., Saito H., Ishii H., Hirohashi S. (2001). Expression of mRNA for DNA methyltransferases and methyl-CpG-binding proteins and DNA methylation status on CpG islands and pericentromeric satellite regions during human hepatocarcinogenesis. *Hepatology*.

[31] Parpart S., Roessler S., Dong F. (2014). Modulation of miR-29 expression by alpha-fetoprotein is linked to the hepatocellular carcinoma epigenome. *Hepatology*.

[32] Zhao Z., Wu Q., Cheng J., Qiu X., Zhang J., Fan H. (2010). Depletion of DNMT3A suppressed cell proliferation and restored PTEN in hepatocellular carcinoma cell. *Journal of Biomedicine and Biotechnology*.

[33] Ding W., Dang H., You H. (2012). miR-200b restoration and DNA methyltransferase inhibitor block lung metastasis of mesenchymal-phenotype hepatocellular carcinoma. *Oncogenesis*.

[34] Sparmann A., van Lohuizen M. (2006). Polycomb silencers control cell fate, development and cancer. *Nature Reviews Cancer*.

[35] Hayashi A., Yamauchi N., Shibahara J. (2014). Concurrent activation of acetylation and tri-methylation of H3K27 in a subset of hepatocellular carcinoma with aggressive behavior. *PLoS ONE*.

[36] He C., Xu J., Zhang J. (2012). High expression of trimethylated histone H3 lysine 4 is associated with poor prognosis in hepatocellular carcinoma. *Human Pathology*.

[37] Cai M.-Y., Hou J.-H., Rao H.-L. (2011). High expression of H3K27me3 in human hepatocellular carcinomas correlates closely with vascular invasion and predicts worse prognosis in patients. *Molecular Medicine*.

[38] Bai X., Wu L., Liang T. (2008). Overexpression of myocyte enhancer factor 2 and histone hyperacetylation in hepatocellular carcinoma. *Journal of Cancer Research and Clinical Oncology*.

[39] Sudo T., Utsunomiya T., Mimori K. (2005). Clinicopathological significance of EZH2 mRNA expression in patients with hepatocellular carcinoma. *British Journal of Cancer*.

[40] Sasaki M., Ikeda H., Itatsu K. (2008). The overexpression of polycomb group proteins Bmi1 and EZH2 is associated with the progression and aggressive biological behavior of hepatocellular carcinoma. *Laboratory Investigation*.

[41] Cai M.-Y., Tong Z.-T., Zheng F. (2011). EZH2 protein: a promising immunomarker for the detection of hepatocellular carcinomas in liver needle biopsies. *Gut*.

[42] Au S. L.-K., Wong C. C.-L., Lee J. M.-F. (2012). Enhancer of zeste homolog 2 epigenetically silences multiple tumor suppressor microRNAs to promote liver cancer metastasis. *Hepatology*.

[43] Wang L., Zhang X., Jia L. T. (2014). C-Myc-mediated epigenetic silencing of MicroRNA-101 contributes to dysregulation of multiple pathways in hepatocellular carcinoma. *Hepatology*.

[44] Cheng A. S. L., Lau S. S., Chen Y. (2011). EZH2-mediated concordant repression of Wnt antagonists promotes *β*-catenin-dependent hepatocarcinogenesis. *Cancer Research*.

[45] Wang W.-H., Studach L. L., Andrisani O. M. (2011). Proteins ZNF198 and SUZ12 are down-regulated in hepatitis B virus (HBV) X protein-mediated hepatocyte transformation and in HBV replication. *Hepatology*.

[46] Studach L. L., Menne S., Cairo S. (2012). Subset of Suz12/PRC2 target genes is activated during hepatitis B virus replication and liver carcinogenesis associated with HBV X protein. *Hepatology*.

[47] Effendi K., Mori T., Komuta M., Masugi Y., Du W., Sakamoto M. (2010). Bmi-1 gene is upregulated in early-stage hepatocellular carcinoma and correlates with ATP-binding cassette transporter B1 expression. *Cancer Science*.

[48] Wang H., Pan K., Zhang H.-K. (2008). Increased polycomb-group oncogene Bmi-1 expression correlates with poor prognosis in hepatocellular carcinoma. *Journal of Cancer Research and Clinical Oncology*.

[49] Liu Z., Liu Y., Wang H. (2009). Patt1, a novel protein acetyltransferase that is highly expressed in liver and downregulated in hepatocellular carcinoma, enhances apoptosis of hepatoma cells. *The International Journal of Biochemistry and Cell Biology*.

[50] Magerl C., Ellinger J., Braunschweig T. (2010). H3K4 dimethylation in hepatocellular carcinoma is rare compared with other hepatobiliary and gastrointestinal carcinomas and correlates with expression of the methylase Ash2 and the demethylase LSD1. *Human Pathology*.

[51] Duong F. H. T., Christen V., Lin S., Heim M. H. (2010). Hepatitis C virus-induced up-regulation of protein phosphatase 2A inhibits histone modification and DNA damage repair. *Hepatology*.

[52] Prensner J. R., Chinnaiyan A. M. (2011). The emergence of lncRNAs in cancer biology. *Cancer Discovery*.

[53] Rinn J. L., Kertesz M., Wang J. K. (2007). Functional demarcation of active and silent chromatin domains in human HOX loci by noncoding RNAs. *Cell*.

[54] He S., Liu S., Zhu H. (2011). The sequence, structure and evolutionary features of HOTAIR in mammals. *BMC Evolutionary Biology*.

[55] Schorderet P., Duboule D. (2011). Structural and functional differences in the long non-coding RNA hotair in mouse and human. *PLoS Genetics*.

[56] Chiyomaru T., Yamamura S., Fukuhara S. (2013). Genistein inhibits prostate cancer cell growth by targeting miR-34a and oncogenic HOTAIR. *PLoS ONE*.

[57] Hermeking H. (2010). The miR-34 family in cancer and apoptosis. *Cell Death and Differentiation*.

[58] Liu X.-H., Sun M., Nie F.-Q. (2014). Lnc RNA HOTAIR functions as a competing endogenous RNA to regulate HER2 expression by sponging miR-331-3p in gastric cancer. *Molecular Cancer*.

[59] Tsai M. C., Manor O., Wan Y. (2010). Long noncoding RNA as modular scaffold of histone modification complexes. *Science*.

[60] Kaneko S., Li G., Son J. (2010). Phosphorylation of the PRC2 component Ezh2 is cell cycle-regulated and up-regulates its binding to ncRNA. *Genes & Development*.

[61] Gupta R. A., Shah N., Wang K. C. (2010). Long non-coding RNA HOTAIR reprograms chromatin state to promote cancer metastasis. *Nature*.

[62] Kogo R., Shimamura T., Mimori K. (2011). Long noncoding RNA HOTAIR regulates polycomb-dependent chromatin modification and is associated with poor prognosis in colorectal cancers. *Cancer Research*.

[63] Nie Y., Liu X., Qu S., Song E., Zou H., Gong C. (2013). Long non-coding RNA HOTAIR is an independent prognostic marker for nasopharyngeal carcinoma progression and survival. *Cancer Science*.

[64] Geng Y. J., Xie S. L., Li Q., Ma J., Wang G. Y. (2011). Large intervening non-coding RNA HOTAIR is associated with hepatocellular carcinoma progression. *The Journal of International Medical Research*.

[65] Yang Z., Zhou L., Wu L.-M. (2011). Overexpression of long non-coding RNA HOTAIR predicts tumor recurrence in hepatocellular carcinoma patients following liver transplantation. *Annals of Surgical Oncology*.

[66] Xu Z.-Y., Yu Q.-M., Du Y.-A. (2013). Knockdown of long non-coding RNA HOTAIR suppresses tumor invasion and reverses epithelial mesenchymal transition in gastric cancer. *International Journal of Biological Sciences*.

[67] Alves C. P., Fonseca A. S., Muys B. R. (2013). Brief report: the lincRNA Hotair is required for epithelial-to-mesenchymal transition and stemness maintenance of cancer cell lines. *Stem Cells*.

[68] Brannan C. I., Dees E. C., Ingram R. S., Tilghman S. M. (1990). The product of the H19 gene may function as an RNA. *Molecular and Cellular Biology*.

[69] Cai X., Cullen B. R. (2007). The imprinted H19 noncoding RNA is a primary microRNA precursor. *RNA*.

[70] Keniry A., Oxley D., Monnier P. (2012). The H19 lincRNA is a developmental reservoir of miR-675 that suppresses growth and Igf1r. *Nature Cell Biology*.

[71] Monnier P., Martinet C., Pontis J., Stancheva I., Ait-Si-Ali S., Dandolo L. (2013). H19 lncRNA controls gene expression of the Imprinted Gene Network by recruiting MBD1. *Proceedings of the National Academy of Sciences of the United States of America*.

[72] Bartolomei M. S., Zemel S., Tilghman S. M. (1991). Parental imprinting of the mouse H19 gene. *Nature*.

[73] Gabory A., Ripoche M. A., Le Digarcher A. (2009). H19 acts as a trans regulator of the imprinted gene network controlling growth in mice. *Development*.

[74] Gabory A., Jammes H., Dandolo L. (2010). The H19 locus: role of an imprinted non-coding RNA in growth and development. *BioEssays*.

[75] Ripoche M.-A., Kress C., Poirier F., Dandolo L. (1997). Deletion of the H19 transcription unit reveals the existence of a putative imprinting control element. *Genes and Development*.

[76] Iizuka N., Oka M., Yamada-Okabe H. (2002). Comparison of gene expression profiles between hepatitis B virus- and hepatitis C virus-infected hepatocellular carcinoma by oligonucleotide microarray data on the basis of a supervised learning method. *Cancer Research*.

[77] Iizuka N., Oka M., Tamesa T., Hamamoto Y., Yamada-Okabe H. (2004). Imbalance in expression levels of insulin-like growth factor 2 and H19 transcripts linked to progression of hepatocellular carcinoma. *Anticancer Research*.

[78] Sohda T., Iwata K., Soejima H., Kamimura S., Shijo H., Yun K. (1998). In situ detection of insulin-like growth factor II (IGF2) and H19 gene expression in hepatocellular carcinoma. *Journal of Human Genetics*.

[79] Zhang L., Yang F., Yuan J.-H. (2013). Epigenetic activation of the MiR-200 family contributes to H19-mediated metastasis suppression in hepatocellular carcinoma. *Carcinogenesis*.

[80] Yang F., Zhang L., Huo X.-S. (2011). Long noncoding RNA high expression in hepatocellular carcinoma facilitates tumor growth through enhancer of zeste homolog 2 in humans. *Hepatology*.

[81] Tripathi V., Ellis J. D., Shen Z. (2010). The nuclear-retained noncoding RNA MALAT1 regulates alternative splicing by modulating SR splicing factor phosphorylation. *Molecular Cell*.

[82] Yang L., Lin C., Liu W. (2011). ncRNA- and Pc2 methylation-dependent gene relocation between nuclear structures mediates gene activation programs. *Cell*.

[83] Kaneko S., Bonasio R., Saldaña-Meyer R. (2014). Interactions between JARID2 and noncoding RNAs regulate PRC2 recruitment to chromatin. *Molecular Cell*.

[84] Wang K. C., Yang Y. W., Liu B. (2011). A long noncoding RNA maintains active chromatin to coordinate homeotic gene expression. *Nature*.

[85] Luo M., Li Z., Wang W., Zeng Y., Liu Z., Qiu J. (2013). Long non-coding RNA H19 increases bladder cancer metastasis by associating with EZH2 and inhibiting E-cadherin expression. *Cancer Letters*.

[86] Matouk I. J., DeGroot N., Mezan S. (2007). The H19 non-coding RNA is essential for human tumor growth. *PLoS ONE*.

[87] Matouk I. J., Raveh E., Abu-lail R. (2014). Oncofetal H19 RNA promotes tumor metastasis. *Biochimica et Biophysica Acta—Molecular Cell Research*.

[88] Tsang W. P., Kwok T. T. (2007). Riboregulator H19 induction of MDR1-associated drug resistance in human hepatocellular carcinoma cells. *Oncogene*.

[89] Yoshimizu T., Miroglio A., Ripoche M.-A. (2008). The H19 locus acts in vivo as a tumor suppressor. *Proceedings of the National Academy of Sciences of the United States of America*.

[90] Lv J., Ma L., Chen X. L., Huang X. H., Wang Q. (2014). Downregulation of LncRNAH19 and MiR-675 promotes migration and invasion of human hepatocellular carcinoma cells through AKT/GSK-3beta/Cdc25A signaling pathway. *Journal of Huazhong University of Science and Technology (Medical Sciences)*.

[91] Lai M.-C., Yang Z., Zhou L. (2012). Long non-coding RNA MALAT-1 overexpression predicts tumor recurrence of hepatocellular carcinoma after liver transplantation. *Medical Oncology*.

[92] Braconi C., Kogure T., Valeri N. (2011). MicroRNA-29 can regulate expression of the long non-coding RNA gene MEG3 in hepatocellular cancer. *Oncogene*.

[93] Quagliata L., Matter M. S., Piscuoglio S. (2014). Long noncoding RNA HOTTIP/HOXA13 expression is associated with disease progression and predicts outcome in hepatocellular carcinoma patients. *Hepatology*.

[94] Guil S., Soler M., Portela A. (2012). Intronic RNAs mediate EZH2 regulation of epigenetic targets. *Nature Structural and Molecular Biology*.

[95] Nakamura K., Aizawa K., Nakabayashi K. (2013). DNA methyltransferase inhibitor zebularine inhibits human hepatic carcinoma cells proliferation and induces apoptosis. *PLoS ONE*.

[96] Andersen J. B., Factor V. M., Marquardt J. U. (2010). An integrated genomic and epigenomic approach predicts therapeutic response to zebularine in human liver cancer. *Science Translational Medicine*.

[97] Tao S. F., Zhang C. S., Guo X. L. (2012). Anti-tumor effect of 5-aza-2′-deoxycytidine by inhibiting telomerase activity in hepatocellular carcinoma cells. *World Journal of Gastroenterology*.

[98] Ma B. B. Y., Sung F., Tao Q. (2010). The preclinical activity of the histone deacetylase inhibitor PXD101 (belinostat) in hepatocellular carcinoma cell lines. *Investigational New Drugs*.

[99] Yeo W., Chung H. C., Chan S. L. (2012). Epigenetic therapy using belinostat for patients with unresectable hepatocellular carcinoma: a multicenter phase I/II study with biomarker and pharmacokinetic analysis of tumors from patients in the Mayo Phase II Consortium and the Cancer Therapeutics Research Group. *Journal of Clinical Oncology*.

